# First application of a droplet digital PCR to detect *Toxoplasma gondii* in mussels

**DOI:** 10.3389/fmicb.2023.1238689

**Published:** 2023-09-08

**Authors:** Andrea Mancusi, Yolande T. R. Proroga, Angela Giordano, Santa Girardi, Francescantonio D’Orilia, Renato Pinto, Paolo Sarnelli, Laura Rinaldi, Federico Capuano, Maria Paola Maurelli

**Affiliations:** ^1^Istituto Zooprofilattico Sperimentale del Mezzogiorno, Portici, Italy; ^2^Centro di Riferimento Regionale Sanità Animale (CReSan), Salerno, Italy; ^3^UOD Prevenzione e Sanità Pubblica Veterinaria Regione Campania, Naples, Italy; ^4^Unit of Parasitology and Parasitic Diseases, Department of Veterinary Medicine and Animal Production, CREMOPAR, University of Naples Federico II, Naples, Italy

**Keywords:** droplet digital polymerase chain reaction, mollusks, mussels, *Toxoplasma gondii*, toxoplasmosis

## Abstract

Toxoplasmosis, caused by the protozoan *Toxoplasma gondii*, is one of the main food-, water- and soil-borne zoonotic disease worldwide. Over the past 20 years many papers were published on the transmission of *T. gondii* by marine animals, including mollusks, which can concentrate the oocysts and release them. Sporulated oocysts may remain viable and infective for 18 months in seawater. Therefore, raw or undercooked bivalve mollusks pose a risk to humans. This study aimed to apply and validate for the first time a very sensitive digital droplet polymerase chain reaction (ddPCR) protocol to detect and quantify *T. gondii* DNA in mussels. Four concentration levels: 8000 genomic copies (gc)/μL, 800 gc/μL, 80 gc/μL, 8 gc/μL of a *T. gondii* reference DNA were tested. DNA was extracted from 80 pools of mussels (*Mytilus galloprovincialis*). Forty pools were contaminated with *T. gondii* reference DNA and used as positive controls, while 40 pools were used as negative controls. DdPCR reaction was prepared using a protocol, previously developed by the authors, for detection of *T. gondii* in meat. Amplification was obtained up 8 gc/μL. All infected replicates resulted positive, as well as no droplets were detected in negative controls. The droplets produced in the reaction ranged from 8,828 to 14,075 (average 12,627 droplets). The sensitivity and specificity of ddPCR were 100% (95%CI = 94.3–99.9). In addition, 100 pools of mussels collected in the Gulf of Naples were used to validate the protocol. Of these 16% were positive (95% CI = 9.7–25.0) for *T. gondii*. Samples were also tested by real-time PCR and no positive samples were found. Data obtained from ddPCR showed good identification of negative and positive samples with higher specificity and efficiency than real-time PCR. This tool could be very useful for a rapid sensitive detection of low DNA concentrations of *T. gondii* in mussels, reducing the risk of toxoplasmosis in humans.

## 1. Introduction

Toxoplasmosis, caused by the protozoan *Toxoplasma gondii*, is one of the main food-, water- and soil-borne zoonotic disease in the world (FAO/WHO, 2014; EFSA Panel on Biological Hazards, 2018; Almeria and Dubey, 2021). The lifecycle of this parasite involves felids (definitive hosts) and all warm-blooded animals and humans (intermediate hosts) (Dubey, 2022; Marín-García et al., 2022). Oocysts, spread into the environment by definitive hosts, become infective after sporulation, representing a risk for intermediate hosts in which sporozoites contained in sporulated oocysts may develop in tachyzoites and tissue cysts (Hatam-Nahavandi et al., 2021). It is estimated that up to 30% of the human world’s population is affected by *T. gondii* (Nayeri et al., 2021). Human transmission can occur congenitally, with the passage of tachyzoites from mother to the fetus (Almeria and Dubey, 2021). Instead, the acquired toxoplasmosis in humans is mainly related to ingestion of raw/undercooked meat of infected animals containing tissue cysts or of other raw foods at risk (e.g., vegetable, fruit, mollusks) or water contaminated by sporulated oocysts (Caradonna et al., 2017; Ghozzi et al., 2017; EFSA European Centre for Disease Prevention and Control, 2021; Dámek et al., 2023). However, few food and waterborne outbreaks have been described worldwide, especially in Europe, as also reported in the last EFSA report (2021) and by Almeria and Dubey (2021). This may be due to several causes, mainly: (i) difficulty connecting infection with food consumption in immunocompetent individuals, where toxoplasmosis appears asymptomatic or mild symptomatic; and (ii) scarce and non-standardized procedures to detect *T. gondii* in food (Almeria and Dubey, 2021; EFSA European Centre for Disease Prevention and Control, 2021; Marín-García et al., 2022).

The meat from different domestic and wild hosts has been investigated as source of *T. gondii* in humans, while environmental contamination is likely understudied and underestimated (Gabriël et al., 2022). In particular, a systematic and metanalysis review (López Ureña et al., 2022) recently published has highlighted that only in the last 20 years have been published papers on the transmission of *T. gondii* by fresh products and marine animals, including mollusks. This is due to increased knowledge also of other food- and water-borne zoonotic protozoa, e.g., *Cryptosporidium* spp., *Giardia duodenalis*, and *Cyclospora cayetanensis* (López Ureña et al., 2022).

Sporulated oocysts may remain viable and infective for 18 months in seawater (Lindsay and Dubey, 2009; Dubey, 2022). Studies on occurrence of *T. gondii* in the aquatic ecosystem started when lethal cases were reported due to this parasite in sea otters in California (Miller et al., 2004). The oocysts eliminated by felids are carried by freshwaters to coastal waters, where they can infect the marine animals (Fayer, 2004). In later years, *T. gondii* was found also in pinnipeds, mustelids and cetaceans, as well as in edible fishs and mollusks (Putignani et al., 2011; Zhang et al., 2014; Marino et al., 2019; Santoro et al., 2020; Nayeri et al., 2021; Dubey, 2022). Bivalve mollusks such as clams, cockles, mussels and oysters filter large volumes of water and can concentrate chemical and biological contaminants, including *T. gondii* oocysts. Moreover, mollusks can also release the parasite with feces after several days of ingestion from the water (Géba et al., 2021; Dubey, 2022). Therefore, raw or undercooked bivalve mollusks pose a risk to humans (Rosário et al., 2021).

Recently, we developed and validated a new droplet digital polymerase chain reaction (ddPCR) very sensitive (95.7%) and specific (100%) to detect *T. gondii* DNA in meat samples. This tool was compared with a real-time PCR protocol, which is usually the most used technique to detect *T. gondii* in food and environmental samples. The ddPCR showed a higher number of positive samples (7.6% of samples analyzed were positive by ddPCR vs 1.2% by qPCR) (Mancusi et al., 2022).

Therefore, in this study we applied and validated for the first time a ddPCR protocol on mussels, to obtain a more sensitive diagnostic tool to detect and quantify *T. gondii* DNA also in these marine animals.

## 2. Materials and methods

### 2.1. DNA extraction and ddPCR performing

To optimize ddPCR detection of *T. gondii*, a specific reference strain from the American Type Culture Collection (ATCC) was used. The *T. gondii* ATCC 50174D contained 2×10^5^ genomic copies (gc)/μL in solution and was diluted with DNAse/RNAse free water to obtain four concentrations: 8000 gc/μL, 800 gc/μL, 80 gc/μL, and 8 gc/μL, to evaluate the amplification limit.

Internal tissue from 80 negative for *T. gondii* pools of marine mussels (*Mytilus galloprovincialis*) were homogenized by a stomacher. Each pool consisted of 10 mussels. The DNA was extracted from 25 mg of homogenized tissue for each pool, using a QIAamp DNA Mini kit (Qiagen, Hilden, Germany), according to the manufacturers’ instructions. Negativity was estimated using the real-time PCR protocol provided by the National Reference Centre for toxoplasmosis (Palermo, Italy). To evaluate the abovementioned concentrations, 40 negative DNA samples were contaminated with DNA extracted from the *T. gondii* ATCC, to obtain 10 replicates for each concentration. To the 40 samples used as negative controls only sterile water was added.

The ddPCR reaction was performed in a QX200 system (Bio-Rad, Hercules, CA, United States), using the protocol (i.e., mix preparation, primers and probe, Table 1) described by Mancusi et al. (2022) to amplify the 529 bp repeat element. Droplets were generated using a DG8 cartridge (Bio-Rad, Hercules, CA, United States), adding a volume of 70 μL of droplet generation oil for each well. Subsequently, the PCR amplification was carried out on a CFX96 instrument (Bio-Rad, Hercules, CA, USA), transferring 40 μL of droplet-partitioned samples to each well and following these thermal conditions: 96°C for 10 min followed by 45 cycles at 98°C for 30 s, 58.5°C for 1 min and a final stage at 98°C for 10 min. After thermocycling, the plate was read in the QX200 Droplet Reader and the QuantaSoft software was used to quantify the DNA target, expressed as the number of genomic copies/1 μL of reaction.

**Table 1:** Primers and probe to amplify the 529 bp repeat element by ddPCR (Mancusi et al., 2022).

	Sequence
Primer forward	CACAGAAGGGACAGAAGT
Primer reverse	TCGCCTTCATCTACAGTC
Probe	FAM-CTCTCCTCCAAGACGGCTGG-BHQ

Moreover, the intra-laboratory repeatability was assessed to verify the robustness of the ddPCR method, calculating the coefficient of variation (CV%) between the assays performed by two operators.

### 2.2. Validation on field samples

**Figure 1:**
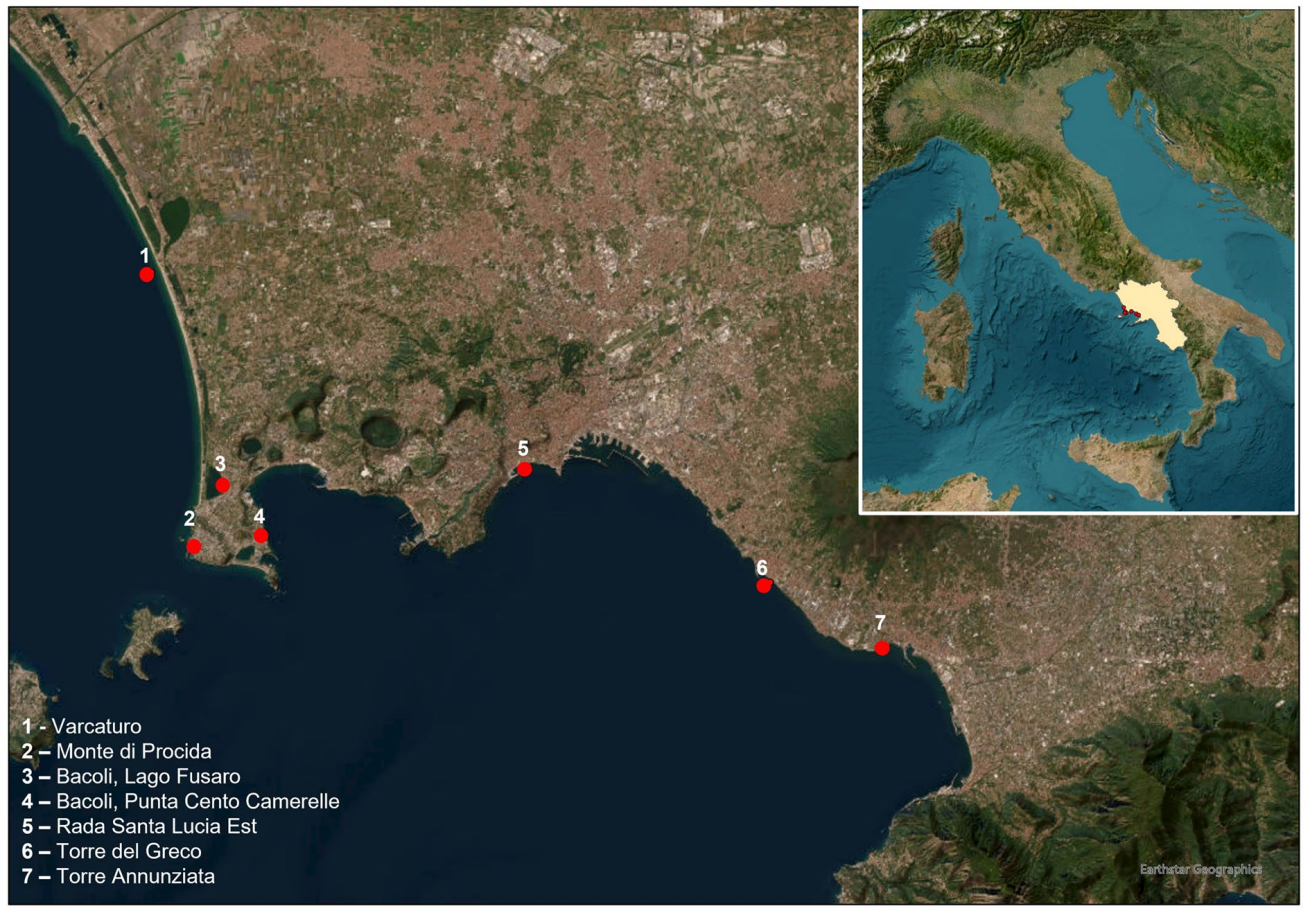
Area of collection of mussels.

One hundred pool samples of marine mussels were collected from seven sites in the Gulf of Naples (Figure 1) and used for validation of the optimized ddPCR. The DNA extraction and ddPCR were performed according to the protocols above mentioned.

These 100 DNA samples were also analyzed by qPCR (Pepe et al., 2021), always amplifying the 529 bp repeat element, to compare the results obtained by ddPCR. Briefly, a final qPCR reaction volume of 20 μL was prepared containing: 1X PCR Master Mix (Bio-Rad, Hercules, CA, United States), 500 nM of each primer (AF1 and AF2 reported in Table 1), 250 nM of TaqMan probe described in Table 1 (Eurofins Genomics, Ebersberg, Germany) and 2 μL of template DNA. Moreover, Internal Amplification controls (IACs) (Applied Biosystems, Waltham, MA, United States), were used to verify the amplification reaction. The amplification conditions were as follows: 50°C for 2 min, 95°C for 10 min, followed by 40 cycles: 95°C for 15 s and 60°C for 1 min. A standard reference curve was prepared, using the four concentrations of *T. gondii* DNA (prepared as mentioned before) in triplicate. The quantified DNA target is expressed as the number of genomic copies/1 μL of reaction. Specificity of primers was evaluated *in silico* using the NCBI nucleotide BLAST tool and in lab using known positive samples for other two protozoa which can be found in mollusks, *Giardia duodenalis* and *Cryptosporidium parvum*.

The Standards for Reporting of Diagnostic Accuracy Studies (STARD) checklist (https://www.equator-network.org/reporting-guidelines/stard/) was used to improve our information on performances of techniques (Cohen et al., 2016).

### 2.3. Statistical analysis

Sensitivity, specificity, negative and positive predictive values (NPV and PPV) were calculated for ddPCR. The agreement between qPCR and ddPCR was calculated using Cohen’s *κ* statistic (Thrusfield, 2007).

The *κ* measure was interpreted as follows: 0, no agreement; 0.01–0.20, poor agreement; 0.21–0.40, fair agreement; 0.41–0.60, moderate agreement; 0.61–0.80, substantial agreement; and 0.81–1.0, nearly perfect agreement (Thrusfield, 2007).

The intra-assay coefficient of variation (CV) for each of the four dilution levels and overall were calculated using the following formula: [(CV% = standard deviation [SD]/mean value for each concentration level) × 100].

The 95% confidence intervals (95% CIs) were calculated using the free online software “Sample Size Calculator” (Creative Research Systems, CA, United States).

## 3. Results

### 3.1. ddPCR performing

**Figure 2:**
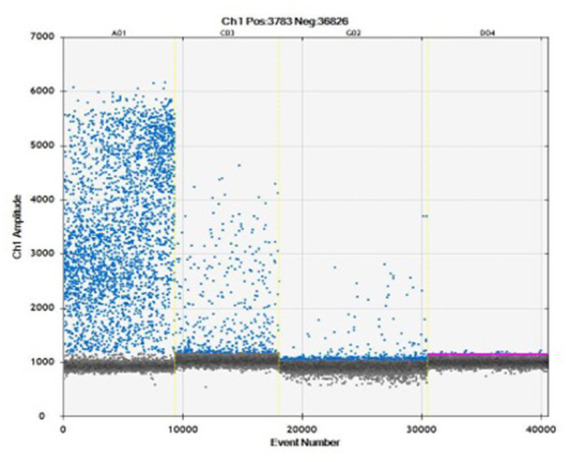
Amplification plot obtained to evaluate four concentrations: 8000 gc/μL (A01), 800 gc/μL (C03), 80 gc/μL (G02), 8 gc/μL (D04) in ddPCR.

The ddPCR allowed DNA amplification up to 8 gc/μL (Figure 2). Samples which showed ≥ two droplets were considered positive. All infected replicates resulted positive, as well as no droplets were detected in negative controls. The droplets produced in the reaction ranged from 8,828 to 14,075 (average 12,627 droplets). The data obtained by ddPCR showed a good separation between negative and positive droplets with few interface droplets, highlighting the high specificity and efficiency of this technique. In fact, the sensitivity and specificity of ddPCR were 100% (95%CI = 94.3–99.9). An overall CV% = 14.8 was calculated for all the ddPCR positive replicates for all the concentration levels. The CV% for each concentration level were: 8000 gc/μL CV% = 7.8, 800 gc/μL CV% = 14.6, 80 gc/μL CV% = 16.6, 8 gc/μL CV% = 20.0.

No significant intra-laboratory variation in results was reported (CV% < 0.1).

### 3.2. Validation of ddPCR

Of the 100 mussel pools (composed by 10 individual samples) examined, the ddPCR detected 16 positive samples (16.0%; 95%CI = 9.7–25.0), with concentrations ranging from 0.1 to 1.9 gc/μL. No positive samples at *T. gondii* were detected by the qPCR reference method. Therefore, no agreement was found between ddPCR and qPCR (*κ* = 0). Samples examined showed no inhibitory effects on qPCR, as demonstrated by the results obtained using the IACs. No cross-reactions with *G. duodenalis* or *C. parvum* amplification were found *in silico* or in lab, showing a specificity of 100% by ddPCR.

All performance of the ddPCR has been summarized in Table 2.

**Table 2:** Performances of ddPCR for *Toxoplasma gondii* detection and quantification.

Performance	ddPCR (%; 95%CI)
Specificity	100; 89.1–99.8
Sensitivity	100; 89.1–99.8
NPV	100; 89.1–99-8
PPV	100; 89.1–99-8

## 4. Discussion

Although, *T. gondii* is one of the most widespread zoonotic foodborne protozoa, no systematic food controls are carried out for this parasite, caused mainly by the lack of specific regulations and standardized methods to detect the parasite in food matrices (Pinto-Ferreira et al., 2019; Gabriël et al., 2022).

Recent outbreaks due to contamination with *T. gondii* oocysts in the environment have highlighted significant risks to public health. Indeed, several studies showed that oocysts are very resistant for months and years in the environment, including the marine environment (López Ureña et al., 2022). Oocysts were found in wild and commercial bivalve mollusks (clams, oysters, and mussels) with detection rates varied between 2.1% (Fung et al., 2021) and 6.6% (Ghozzi et al., 2017) in clams from Canada and Tunisia, respectively; from 1.3% (Silva et al., 2020) to 31.0% (Marquis et al., 2019) in oysters from Brazil and United States, respectively; from 1.4% (Shapiro et al., 2015) to 46.3% (Staggs et al., 2015) in different species of mussels from United States (López Ureña et al., 2022). *T gondii* DNA has been found also in different fish species ranging from 2.9 to 100% (depending on fish species; Marino et al., 2019), in sea otters (54.8%) and cetaceans (30.9%) (Ahmadpour et al., 2022).

Unfortunately, it is not easy to compare different studies for the detection of *T. gondii* in marine animals including mollusks, because several molecular methods and protocols are used (i.e., singleplex PCR, (semi-)nested, PCR–restriction fragment length polymorphism, qPCR), characterized by different performance (sensitivity, specificity, accuracy, precision, repeatability, detection limit) (Nayeri et al., 2021; Marín-García et al., 2022). The most common targets employed are the B1 gene or the 529 bp DNA repeat element which are multi-copy loci that increase the sensitivity of *T. gondii* detection (López Ureña et al., 2022). In particular, the 529 bp repeat element resulted more sensitive and accurate of B1 also at low concentrations of *T. gondii* DNA (Edvisson et al., 2006; Sağlam et al., 2021). However, more performant tools are in continuous development. In this study better results were obtained by ddPCR than qPCR (16.0% of positive samples for *T. gondii* obtained by ddPCR vs 0% by qPCR), confirming that this technique is very useful to increase the sensitivity, accuracy and precision for detection and quantification of small amounts of DNA of the pathogens to be recognized, preserving all the advantages of the qPCR (Kao et al., 2021). This ddPCR protocol was previously successfully used for the detection of *T. gondii* in meat, showing 7.6% of positive sample vs 1.2% by qPCR (Mancusi et al., 2022).

For these reasons, the ddPCR could be very useful for a rapid sensitive detection of low DNA concentrations of *T. gondii,* in order to perform a standardized food inspection on several matrices, reducing the public health risk of this parasite.

However, our preliminary results need to be confirmed by further studies on field samples.

Finally, this study confirms the presence of *T. gondii* in mussels in Campania region, as previously showed by Santoro et al., 2020, but the prevalence of *T. gondii* found in our study in mussels collected in Gulf of Naples is higher (16% vs 10.2% in Santoro et al., 2020). Moreover, this occurrence is higher also than other previous studies on clams, mussels and oyster collected in other two Italian regions: Apulia and Sardinia (Putignani et al., 2011; Tedde et al., 2019), highlighting the need of ministerial regulations to prevent toxoplasmosis infection.

## Data availability statement

The raw data supporting the conclusions of this article will be made available by the authors, without undue reservation.

## Ethics statement

The manuscript presents research on animals that do not require ethical approval for their study.

## Author contributions

AM, YP, AG, and SG performed sampling and laboratory analyses. AM, LR, FC, and MM conceived the study. FD’O, RP, PS, LR, FC, and MM supervised the study. MM and AM wrote the manuscript. All authors contributed to manuscript revision, read and approved the submitted version.

## Funding

This study was supported by the Regional Project “Control and reduction of toxoplasmosis in animals and humans – ToxoCamp,” Campania Region, Italy.

## Conflict of interest

The authors declare that the research was conducted in the absence of any commercial or financial relationships that could be construed as a potential conflict of interest.

## Publisher’s note

All claims expressed in this article are solely those of the authors and do not necessarily represent those of their affiliated organizations, or those of the publisher, the editors and the reviewers. Any product that may be evaluated in this article, or claim that may be made by its manufacturer, is not guaranteed or endorsed by the publisher.
